# Cost-Effectiveness Analysis of Nivolumab Plus Ipilimumab vs. Chemotherapy as First-Line Therapy in Advanced Non-Small Cell Lung Cancer

**DOI:** 10.3389/fonc.2020.01649

**Published:** 2020-09-08

**Authors:** Huabin Hu, Longjiang She, Mengting Liao, Yin Shi, Linli Yao, Dong Ding, Youwen Zhu, Shan Zeng, David P. Carbone, Jin Huang

**Affiliations:** ^1^Department of Medical Oncology, The Sixth Affiliated Hospital of Sun Yat-sen University, Guangzhou, China; ^2^Guangdong Provincial Key Laboratory of Colorectal and Pelvic Floor Diseases, Guangdong Institute of Gastroenterology, Guangzhou, China; ^3^Department of Oncology, Xiangya Hospital, Central South University, Changsha, China; ^4^Xiangya Hospital, Central South University, Changsha, China; ^5^Department of Pharmacy, Xiangya Hospital, Central South University, Changsha, China; ^6^Barbara J. Bonner Chair in Lung Cancer Research, James Thoracic Center, James Cancer Center, The Ohio State University Medical Center, Columbus, OH, United States

**Keywords:** cost-effectiveness, nivolumab, ipilimumab, chemotherapy, non-small cell lung cancer

## Abstract

**Background:** The CheckMate 227 trial has indicated that nivolumab plus ipilimumab compared with chemotherapy significantly increases long-term survival in the first-line setting of advanced non-small-cell lung cancer (NSCLC).

**Methods:** A Markov model was built to estimate the cost and effectiveness of nivolumab plus ipilimumab vs. chemotherapy as the first-line therapy in patients with advanced NSCLC based on outcomes data from the CheckMate 227 trial. We calculated the cost and health outcomes at a willingness-to-pay (WTP) threshold of $150,000 per quality adjusted life year (QALY) in populations with different programmed death ligand 1 (PD-L1) expression levels (≥50, ≥1, and <1%) or a high tumor mutational burden (TMB) (≥10 mutations per megabase). Sensitivity analysis were used to test the model stability.

**Results:** The outcomes showed that the incremental costs and QALYs by using nivolumab plus ipilimumab were $124180.76 and 1.16, $70951.42 and 0.53, $144093.63 and 0.83 for the advanced NSCLC patients with a PD-L1 expression ≥50%, ≥1%, and <1%, which led to an incremental cost-effective ratio (ICER) of $107403.72, $133732.20, and $172589.15 per QALY, respectively. For patients with a high TMB, nivolumab plus ipilimumab contributed an extra 2.04 QALYs at a cost of $69182.50 per QALY.

**Conclusion:** Nivolumab plus ipilimumab as first-line therapy makes a better cost-effective strategy than chemotherapy in advanced NSCLC patients with PD-L1 expression levels ≥50% and ≥1% or a high TMB, at a willingness-to-pay threshold of $150,000 per QALY, but not in the patients with a PD-L1 expression <1%.

## Introduction

Around the globe, lung cancer is the leading cause of cancer incidence and mortality, with 2.1 million new lung cancer cases and 1.8 million deaths worldwide (1–4). Up to 61% of patients with NSCLC had advanced disease at the time of diagnosis, with a 5-years survival rate of 18% (5, 6). Platinum-based chemotherapy doublet or pembrolizumab monotherapy for patients with a high level of tumor PD-L1 expression (≥1%) were the standard first-line therapy for advanced NSCLC without treatable driver mutations (7–10).

Nivolumab, the fully human immunoglobulin G4 monoclonal antibody inhibitor of programmed death-1 (PD-1), and ipilimumab, a fully human immunoglobulin G1 monoclonal antibody that targets the cytotoxic T-cell lymphocyte antigen-4 (CTLA-4) checkpoint receptor, are immune checkpoint inhibitors with distinct but complementary mechanisms of action. In preclinical and clinical settings, the combination of nivolumab plus ipilimumab has presented enhanced activity over nivolumab monotherapy's, which has been approved for the treatment of metastatic melanoma and renal-cell carcinoma (11–16). In the pivotal phase three trial CheckMate 227, the first-line therapy using nivolumab plus ipilimumab brought about a longer duration of overall survival (OS) than that of patients with advanced NSCLC using chemotherapy, regardless of PD-L1 expression levels (17, 18). Nivolumab plus ipilimumab was subsequently approved as the first-line treatment for patients with metastatic NSCLC, PD-L1 ≥ 1%, without EGFR or ALK genomic tumor aberrations by the United States (US) Food and Drug Administration (FDA) in May, 2020.

To our knowledge, it is still unclear whether the use of first-line nivolumab and ipilimumab would be cost-effective for advanced NSCLC with different PD-L1 expression levels. This study aims to evaluate the cost-effectiveness of nivolumab plus ipilimumab vs. chemotherapy in previous untreated advanced NSCLC patients without driver alterations that can be targeted. The cost-effectiveness analyses were conducted, respectively, in three populations with different PD-L1 expression levels (≥50, ≥1, and <1%) or patients with a high tumor mutational burden (TMB) (≥10 mutations per megabase), using the most recently reported data from CheckMate 227 (17–19).

## Materials and Methods

### Model Structure

A Markov model was constructed on the basis of outcomes data from the CheckMate 227 trial to evaluate the costs and effectiveness of using nivolumab plus ipilimumab vs. chemotherapy as first-line therapy for advanced NSCLC from the US payer's perspective. The Markov model cycle length was 6-weeks and the time horizon were 20-years. We adopted a 3% discount rate per year for both costs and outcomes (20). The total costs, life years (LYs), quality adjusted life years (QALYs), and incremental cost-effective ratios (ICERs) were calculated in each treatment strategy. The Markov model was constructed via TreeAge Pro 2018 (TreeAge Software Inc., Williamstown, MA).

The model structure included three states to represent the progression of advanced NSCLC: progression-free survival (PFS), progressive disease (PD), and death (Supplementary Figure 1). Patients were treated with nivolumab plus ipilimumab or chemotherapy in the PFS state until progression. All patients could continue subsequent treatment until death if any disease progression or unacceptable toxic effects occurred. Grades 3 or 4 adverse events (AEs) with a ≥1% frequency reported in CheckMate 227 trial were included.

### Model Survival and Progression Risk Estimates

The estimates of OS for the nivolumab plus ipilimumab group and for the chemotherapy group were based on the OS curves from CheckMate 227 trial. The GetData Graph Digitizer (version 2.25; http://www.getdata-graph-digitizer.com/index.php) was applied to extracting the data points from the OS Kaplan-Meier curves reported in the CheckMate 227 trial, and these data points were then used to fit parametric survival models. The Weibull survival curves matched the number of patients in three states including PFS, PD and death overtime, as the Weibull distribution was flexible and widely used in cancer survival analysis according to Akaike information criterion. Then, we estimated the shape parameter (γ) and the scale parameter (λ) from this fit, and applied Kaplan-Meier curves by using R software package (http://www.r-project.org) and the method of Hoyle et al. (21). With the mean OS time denoted as S(t), the cause-specific mortality M at cycle t can be computed:

(1) $$\begin{array}{l} \text{M}=\frac{\text{S}\left(t\right)-S\left(t\;+\;1\right)}{S\left(t\right)}, \end{array}$$

while S(*t*) = exp(−λ*t*^γ^) (λ > 0; γ > 0).

Finally, OS rates in each cycle were:

1-exp (Scale^*^(_stage) ∧Shape-Scale^*^(_stage+1) ∧Shape)

The progression risks for nivolumab plus ipilimumab group and chemotherapy group were estimated by the same approach. We used this measure to evaluate the OS rate and PFS rate for two groups, that is, patients with three PD-L1 expression levels (≥50, ≥1, and <1%) and those with a high TMB (≥10 mutations per megabase).

### Utility Estimates

Utility was adopted to measure patient's preference for living at a particular health state that is often referred to as QALYs (0 stood for death and 1 for perfect heath), which reflected the impacts of the disease-related health states. We used utilities of 0.784 and 0.693 for the patients with nivolumab plus ipilimumab and chemotherapy as the first-line therapy, respectively, based on the patient-reported outcomes results from CheckMate 227 trial (19). The previously published utility of 0.473 for the NSCLC patients receiving subsequent treatment was used (22).

### Cost Inputs

This study only takes into account direct medical costs, included drug, radiographic examination, administration and AEs costs. The patients in nivolumab plus ipilimumab group were treated with nivolumab (3 mg per kilogram of body weight every 2 weeks) plus ipilimumab (1 mg per kilogram every 6 weeks). The chemotherapy group were treated with platinum-doublet chemotherapy every 3 weeks for up to four cycles{non-squamous NSCLC were treated with pemetrexed (500 mg/m^2^ of body surface area) plus cisplatin (75 mg/m^2^) or carboplatin (area under the concentration-time curve [AUC], 5 or 6), and for squamous NSCLC, with gemcitabine (1,000 or 1,250 mg/m^2^) plus cisplatin (75 mg/m^2^) or gemcitabine (1,000 mg/m^2^) plus carboplatin (AUC, 5)}(17, 18). After four cycles of platinum-doublet chemotherapy, patients with non-squamous NSCLC were received pemetrexed as maintenance therapy (500 mg/m^2^ every 3 weeks) (Table 1 and Supplementary Table 3).

**Table 1:** Model parameters: baseline values, ranges, and distributions for sensitivity analysis in patients with different PD-L1 expression (≥50, ≥1, and <1%).

**Variable**	**Baseline value**	**Range**		**Reference**	**Distribution**
		**Minimum**	**Maximum**		
**Weibull survival model in nivolumab plus ipilimumab group with PD-L1** **≥** **50%**					
PFS	Shape = 0.53605, Scale = 0.27369	-	-	(17)	-
OS	Shape = 0.658678, Scale = 0.112585	-	-	(17)	-
**Weibull survival model in chemotherapy group with PD-L1** **≥** **50%**					
PFS	Shape = 1.10045, Scale = 0.17421	-	-	(17)	-
OS	Shape = 0.868245, Scale = 0.093252	-	-	(17)	-
**Weibull survival model in nivolumab plus ipilimumab group with PD-L1** **≥** **1%**					
PFS	Shape = 0.51890, Scale = 0.36477	-	-	(17)	-
OS	Shape = 0.79585, Scale = 0.09415	-	-	(17)	-
**Weibull survival model in chemotherapy group with PD-L1** **≥** **1%**					
PFS	Shape = 0.97117, Scale = 0.21656	-	-	(17)	-
OS	Shape = 0.97371, Scale = 0.07182	-	-	(17)	-
**Weibull survival model in nivolumab plus ipilimumab group with PD-L1** **<** **1%**					
PFS	Shape = 0.66247, Scale = 0.29319	-	-	(17)	-
OS	Shape = 0.764844, Scale = 0.098260	-	-	(17)	-
**Weibull survival model in chemotherapy group with PD-L1** **<** **1%**					
PFS	Shape = 1.19322, Scale=0.16572	-	-	(17)	-
OS	Shape = 1.023457, Scale = 0.085114	-	-	(17)	-
**Proportion of tumor histologic type in chemotherapy PD-L1** **≥** **50% and** **≥1%**					
Non-squamous	70.8	-	-	(17)	-
Squamous	29.2	-	-	(17)	-
**PD-L1** **<** **1%**					
Non-squamous	75.3	-	-	(17)	-
Squamous	24.7	-	-	(17)	-
**Proportion of treatment discontinuation PD-L1** **≥** **50% and** **≥1%**					
Nivolumab plus ipilimumab	0.422	-	-	(17)	-
Chemotherapy	0.625	-	-	(17)	-
**PD-L1** **<** **1%**					
Nivolumab plus ipilimumab	0.519	-	-	(17)	-
Chemotherapy	0.570	-	-	(17)	-
**Risk for main adverse events in nivolumab plus ipilimumab group with PD-L1** **≥** **50% and** **≥1%**					
Risk of rash	0.023	0.0184	0.0276	(17)	
Risk of diarrhea	0.015	0.012	0.018	(17)	
Risk of fatigue	0.02	0.016	0.024	(17)	
Risk of decreased appetite	0.01	0.008	0.012	(17)	
Risk of anemia	0.013	0.0104	0.0156	(17)	
**Risk for main adverse events in chemotherapy group with PD-L1** **≥** **50% and** **≥1%**					
Risk of anemia	0.106	0.0848	0.1272	(17)	Beta
Risk of neutropenia	0.07	0.056	0.084	(17)	Beta
Risk of neutrophil count decreased	0.085	0.068	0.102	(17)	Beta
Risk of nausea	0.018	0.0144	0.0216	(17)	Beta
Risk of fatigue	0.01	0.008	0.012	(17)	Beta
Risk of decreased appetite	0.01	0.008	0.012	(17)	Beta
Risk of vomiting	0.026	0.0208	0.0312	(17)	Beta
**Risk for main adverse events in nivolumab plus ipilimumab group with PD-L1** **<** **1%**					
Risk of diarrhea	0.022	0.0176	0.0264	(17)	Beta
Risk of fatigue	0.011	0.0088	0.0132	(17)	Beta
Risk of anemia	0.137	0.1096	0.1644	(17)	Beta
**Risk for main adverse events in chemotherapy group with PD-L1** **<** **1%**					
Risk of anemia	0.137	0.1096	0.1644	(17)	Beta
Risk of neutropenia	0.115	0.092	0.138	(17)	Beta
Risk of nausea	0.027	0.0216	0.0324	(17)	Beta
Risk of fatigue	0.022	0.0176	0.0264	(17)	Beta
Risk of decreased appetite	0.016	0.0128	0.0192	(17)	Beta
Risk of vomiting	0.016	0.0128	0.0192	(17)	Beta
Risk of diarrhea	0.011	0.0088	0.0132	(17)	Beta
**Nivolumab plus ipilimumab group subsequent therapy proportion in PD-L1** **≥** **50% and** **≥1% population**					
Radiotherapy	0.174	-	-	(17)	-
Chemotherapy	0.316	-	-	(17)	-
Post-study nivolumab	0.04	-	-	(17)	-
Targeted therapy	0.053	-	-	(17)	-
**Chemotherapy group subsequent therapy proportion in PD-L1** **≥** **50% and** **≥1% population**					
Radiotherapy	0.244	-	-	(17)	-
Chemotherapy	0.275	-	-	(17)	-
Post-study nivolumab	0.325	-	-	(17)	-
Pembrolizumab	0.081	-	-	(17)	-
Targeted therapy	0.043	-	-	(17)	-
**Nivolumab plus ipilimumab group subsequent therapy proportion in PD-L1** **<** **1% population**					
Radiotherapy	0.182	-	-	(17)	-
Chemotherapy	0.422	-	-	(17)	-
Targeted therapy	0.064	-	-	(17)	-
**Chemotherapy group subsequent therapy proportion in PD-L1** **<** **1% population**					
Radiotherapy	0.167	-	-	(17)	-
Chemotherapy	0.344	-	-	(17)	-
Post-study nivolumab	0.301	-	-	(17)	-
Targeted therapy	0.091	-	-	(17)	-
**Utility**					
Utility PFS in nivolumab plus ipilimumab	0.784	0.74	0.828	(19)	Beta
Utility PFS in chemotherapy	0.693	0.642	0.743	(19)	Beta
Utility progressive disease	0.473	0.166	0.568	(22)	Beta
Patients' weight, kg	70	-	-	(23)	Beta
Body surface area, m^2^	1.84	-	-	(23)	Beta
**Drug cost, $/per cycle**					
Nivolumab	17517.15	14013.72	21020.58	(17, 24)	Gamma
Ipilimumab	10718.96	8575.17	12862.75	(17, 24)	Gamma
Pemetrexed	12782.85	10226.28	15339.42	(17, 24)	Gamma
Gemcitabine	92	73.6	110.4	(17, 24)	Gamma
Carboplatin	56.55	45.24	67.86	(17, 24)	Gamma
Cisplatin	51.78	41.42	62.14	(17, 24)	Gamma
Pembrolizumab	19755.6	15804.48?	23706.72?	(17, 24)	Gamma
Post-study nivolumab	17517.15	14013.72	21020.58	(17, 24)	Gamma
Radiotherapy	15899.24	12719.39	19079.09	(25)	Gamma
Targeted therapy	12615.68	10092.54	15138.82	(17, 26)	Gamma
Subsequent chemotherapy	238.74	190.99	286.49	(17, 24)	Gamma
**Expenditures on main adverse events, $**					
Anemia	7969.56	6375.65	9536.47	(27)	Gamma
Neutropenia	32,995	24,746	41,244	(28)	Gamma
Neutrophil count decreased	32,995	24,746	41,244	(28)	Gamma
Fatigue	0	-	-	(29)	Gamma
Rash	13,376	10700.8	16051.2	(30)	Gamma
Diarrhea	10,301	8240.8	12361.2	(30)	Gamma
Decreased appetite	9,711	7768.8	11653.2	(30)	Gamma
Vomiting	10,301	8240.8	12361.2	(30)	Gamma
Nausea	10,301	8240.8	12361.2	(30)	Gamma
Administration $/per cycle	139.61	111.69	167.53	(31)	Gamma
CT $/per cycle	231	208	254	(32)	Gamma
Laboratory $/per cycle	315	252	378	(33)	Gamma
Discount rate	0.03	-	-	(20)	-

*CT, compute tomography; NSCLC, non-small-cell lung cancer; OS, overall survival; PD-L1, programmed death ligand 1; PFS, progression-free survival*.

We used a standard AUC, 6 mg/mL/min, and assumed male sex, 65 years old, body weight, 70-kg, height, 178-cm, body surface area, 1.84 m^2^, and serum creatinine, 1 (23). The price was derived from the Centers for Medicare & Medicaid Services and published articles, and the details were demonstrated in Table 1 and Supplementary Table 3 (24–26, 31–33). The costs of radiographic examination covered computed tomography (CT) (every 6 weeks after treatment and every 9 weeks after progression) (32). Grade 3 or higher AEs with a frequency of >1% were included. The costs related to AEs were calculated by multiplying the incidence of serious AEs by the costs of managing serious AEs per event. AEs costs were derived from previously published studies (27–30). All information regarding the drugs dose, costs were listed in Table 1 and Supplementary Table 3.

### Sensitivity Analysis

One-way sensitivity analyses were performed to test the corresponding ICERs by varying each input parameter within a plausible range, as shown in Table 1. One thousand Monte Carlo simulations were performed to conduct the probabilistic sensitivity analysis by inputting values drawn from their statistical distributions. The probabilistic sensitivity analysis were conducted to estimate the probability of nivolumab plus ipilimumab being cost-effective compared with platinum-doublet chemotherapy in three populations with PD-L1 expression ≥50, ≥1, and <1% and in patients with high TMB at a willing-to-pay (WTP) threshold of $150,000 (31).

## Results

### Base Case Results

In the first-line setting of advanced NSCLC patients without driver alterations that can be targeted, nivolumab plus ipilimumab dwarfed chemotherapy with an additional 1.83 LYs, 0.71 Lys, and 1.37 LYs in the PD-L1 expression ≥50, ≥1, and <1% populations, respectively. When compared to chemotherapy, the mean incremental costs and QALYs of nivolumab plus ipilimumab were $124180.76 and 1.16, $70951.42 and 0.53, $144093.63 and 0.83 for the patients with a PD-L1 expression ≥50, ≥1, and <1%, respectively. Resulting an ICERs of $107403.72 per QALY in PD-L1 ≥50% population, $133732.20 in PD-L1 ≥1% population and $172589.15 in PD-L1 <1% population (Table 2). For patients with a high TMB, the use of nivolumab plus ipilimumab cost an additional $141255.72, provided an additional 2.04 QALYs and an ICER of $69182.50 per QALY compared with chemotherapy (Supplementary Table 4).

**Table 2:** Baseline results in nivolumab plus ipilimumab and chemotherapy groups in PD-L1 expression ≥50, ≥1, and <1% populations.

**Strategies and scenarios**	**Total cost, $**	**LYs**	**QALYs**	**ICER per LY ^**a**^**	**ICER per QALY ^**b**^**
**PD-L1** **≥** **50%**					
Nivolumab plus ipilimumab	390218.01	4.69	2.81	67925.33	107403.72
Chemotherapy	266037.25	2.87	1.66	-	-
**PD-L1** **≥** **1%**					
Nivolumab plus ipilimumab	318368.06	3.43	2.13	100527.95	133732.20
Chemotherapy	247416.64	2.73	1.60	-	-
**PD-L1** **<** **1%**					
Nivolumab plus ipilimumab	314172.48	3.63	2.20	105334.45	172589.15
Chemotherapy	170078.85	2.26	1.36	-	-

*a, Compared to chemotherapy ($/LY); b, Compared to chemotherapy ($/QALY)*.

*ICER, incremental cost-effectiveness ratio; LY, life-year; PD-L1, programmed death ligand 1; QALY, quality-adjusted life-year*.

### Sensitivity Analysis

**Figure 1:**
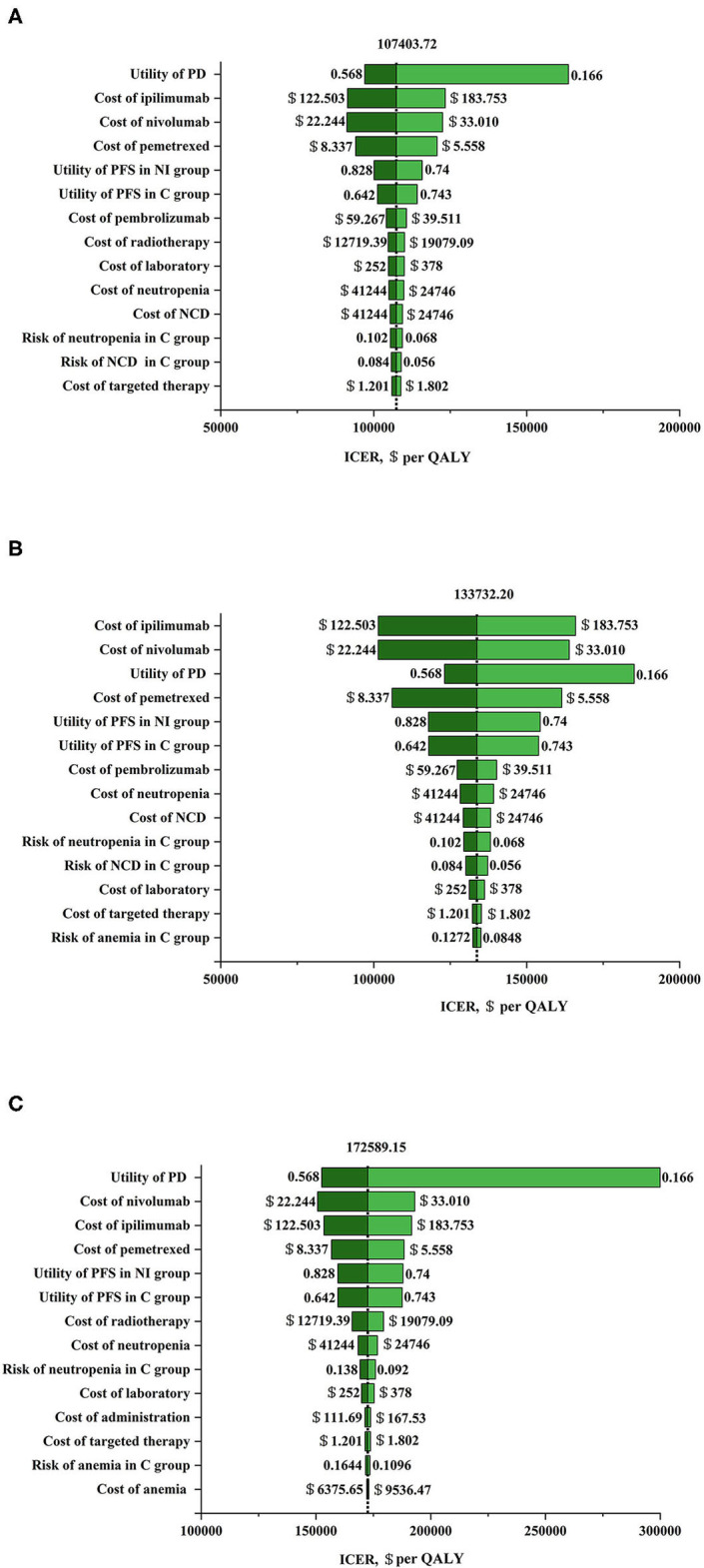
Tornado diagram for one-way sensitivity analysis. **(A)** Nivolumab plus ipilimumab vs. chemotherapy in programmed death ligand 1 (PD-L1) ≥ 50% population. The parameters tested in this one-way sensitivity analysis were displayed in the right of the figure. The vertical dotted line represents incremental cost-effective ratio (ICER) $107403.72/ quality adjusted life year (QALY) (the results of baseline analysis). **(B)** Nivolumab plus ipilimumab vs. chemotherapy in PD-L1 ≥ 1% population. The parameters tested in this one-way sensitivity analysis were displayed in the right of the figure. The vertical dotted line represents ICER $133732.20/QALY (the results of baseline analysis). **(C)** Nivolumab plus ipilimumab vs. chemotherapy in PD-L1 <1% population. The parameters tested in this one-way sensitivity analysis were displayed in the right of the figure. The vertical dotted line represents ICER $172589.15/QALY (the results of baseline analysis). C group, chemotherapy group; NCD, neutrophil count decreased; NI group, nivolumab plus ipilimumab group; PD, progressive disease; PFS, progression-free survival.

The results of univariable sensitivity analysis in populations with three PD-L1 expression levels were showed in Figure 1. The cost of nivolumab plus ipilimumab and the utility of PD possessed the greatest influences on ICERs, which were similar in three populations with PD-L1 expression ≥50, ≥1, and <1%. Nivolumab plus ipilimumab as first-line therapy in advanced NSCLC can be cost-effective compared with chemotherapy if the cost of nivolumab was cut by 21% or the cost of ipilimumab down by 24% in patients with a PD-L1 expression <1% at a WTP threshold of $150,000 per QALY.

**Figure 2:**
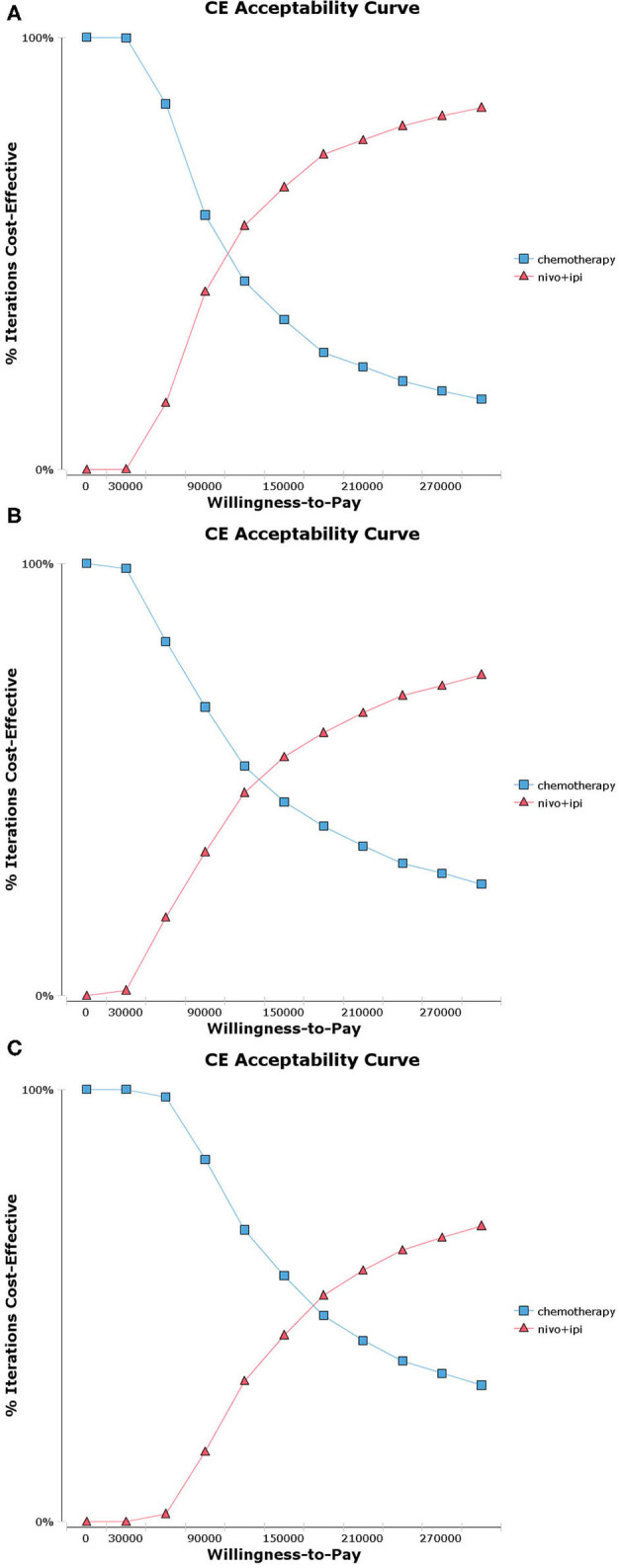
Acceptability curves for the choice of nivolumab plus ipilimumab and chemotherapy treatment strategies at different willingness-to-pay (WTP) thresholds in patients with advanced NSCLC. **(A)** Nivolumab plus ipilimumab vs. chemotherapy in programmed death ligand 1 (PD-L1) ≥ 50% population. **(B)** Nivolumab plus ipilimumab vs. chemotherapy in PD-L1 ≥ 1% population. **(C)** Nivolumab plus ipilimumab vs. chemotherapy in PD-L1 <1% population.

**Figure 3:**
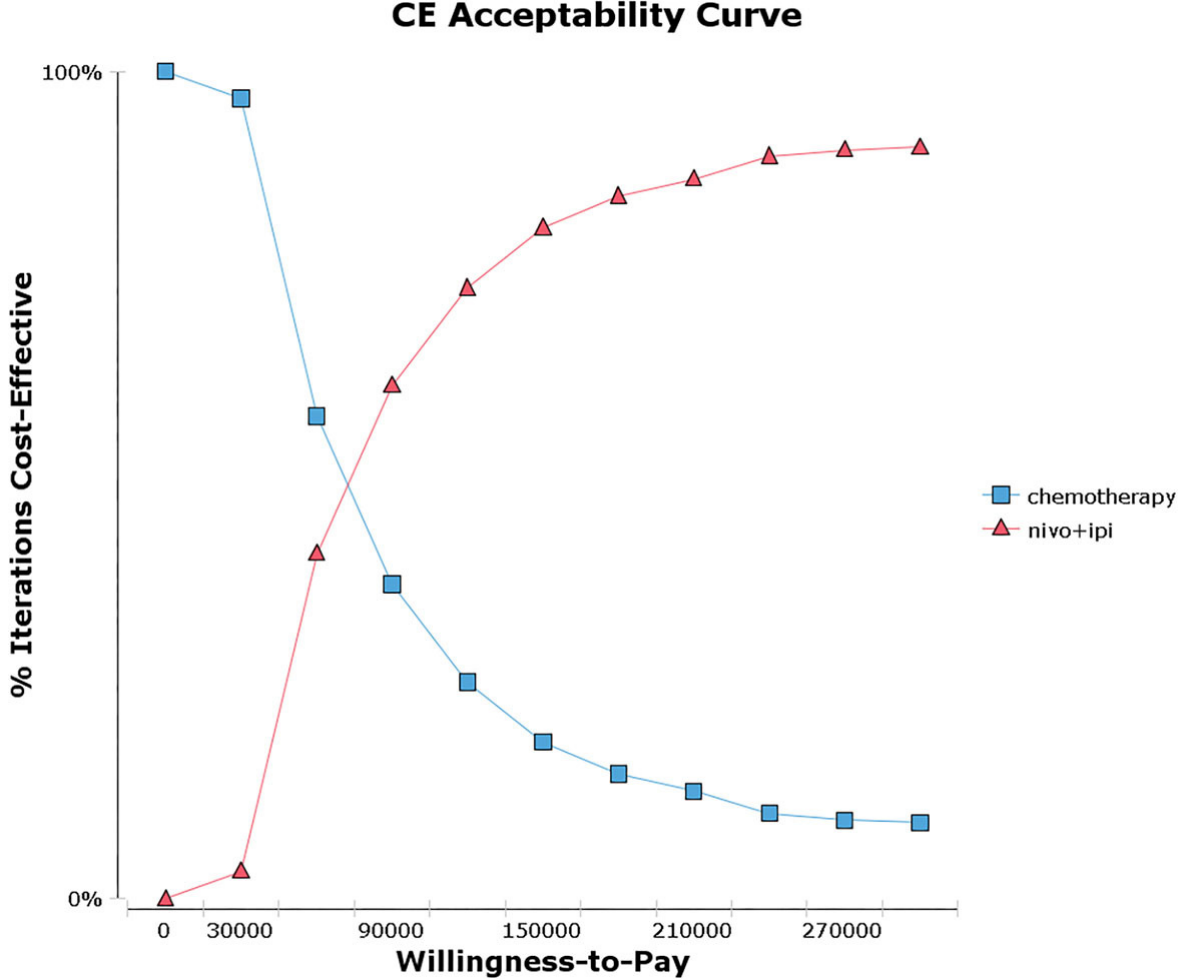
Acceptability curves for the choice of nivolumab plus ipilimumab and chemotherapy treatment strategies at different willingness-to-pay (WTP) thresholds in patients with advanced NSCLC and high tumor mutational burden (TMB).

Probabilistic sensitivity analysis suggested that, compared with chemotherapy, nivolumab plus ipilimumab yield 65.3%, 55.2%, and 43.1% probability of cost-effectiveness at a WTP threshold of $150,000 per QALY for patients with a PD-L1 ≥50%, ≥1% and <1% respectively (Figure 2 and Supplementary Figure 2). There was an 81.1% chance that nivolumab plus ipilimumab was cost-effective for patients with a high TMB (Figure 3 and Supplementary Figure 3).

As the results of the subgroup analyses demonstrated, the ICER of nivolumab plus ipilimumab could be most cost-effective for patients with male, squamous histologic type, bone or central nervous system metastases, regardless of PD-L1 expression levels (Supplementary Tables 1, 2).

## Discussion

The phase 3 study CheckMate 227 was the first trial that showed positive results in dual checkpoint inhibition (anti-CTLA-4 and PD-1) in the field of lung cancer. Previous studies suggested that combination immunotherapy with nivolumab plus ipilimumab could be considered a cost-effect choice in intermediate- and poor-risk patients with metastatic renal cell carcinoma in the US (31, 34). However, it is unclear whether treatment with nivolumab plus ipilimumab as first-line therapy for patients with advanced NSCLC is cost-effective.

The current study is the first cost-effectiveness analysis of nivolumab plus ipilimumab vs. chemotherapy in previously untreated advanced NSCLC patients with different PD-L1 expressions (≥50, ≥1, and <1%) and a high TMB. Case-based results indicated that the ICERs of nivolumab plus ipilimumab vs. chemotherapy were $107403.72, $133732.20, and $172589.15 per additional QALY in PD-L1 ≥50, ≥1, and <1% populations, respectively. The one-way sensitivity analyses revealed that the cost of nivolumab plus ipilimumab and the utility value of PD were the greatest influence factors in all PD-L1 populations. The probabilistic sensitivity analyses depicted a high likelihood that nivolumab plus ipilimumab would be considered a cost-effective choice at a WTP threshold of $150,000 per QALY in the PD-L1 ≥50 and 1% populations, whereas it is not cost-effective in the PD-L1 <1% populations. Further analysis indicated that the nivolumab plus ipilimumab strategy would be cost-effective at a WTP threshold of $150,000 per QALY for PD-L1 <1% populations by reducing the cost of nivolumab or ipilimumab. In addition, irrespective of tumor PD-L1 expression levels, the ICER of nivolumab plus ipilimumab vs. chemotherapy was $69182.50 for high TMB populations. On the other hand, the results of subgroup analysis exhibited that the ICER of nivolumab plus ipilimumab could be improved by selecting patients in accordance with clinical and pathological parameters (Supplementary Tables 1, 2).

In history, PD-L1 expression has been regarded as a major biomarker of response to PD-1 and PD-L1 inhibitors in light of mechanism of action. The outcomings of a *post hoc* analysis from CheckMate 026, nonetheless, implied that the application of TMB as a predictive biomarker instead of or in addition to PD-L1 expression may be conducive to selecting patients with advanced NSCLC who embrace great possibility of reaping benefits of immunotherapy (35). TMB is an emerging biomarker of immunotherapy outcomes for lung cancer (35–39). The results of CheckMate 568 showed the TMB of more than 10 mutations per megabase could be used as an effective cutoff value for selecting the most likely responding patients (40). It was observed in clinical experience that tumor PD-L1 expression and TMB had no significant correlation between the two biomarkers. Similarly, the analysis results obtained by Hellmann MD et al. attested that first-line treatment with nivolumab plus ipilimumab provided clinical benefits for patients with NSCLC and a high TMB (≥10 mutations per megabase), regardless of their tumor PD-L1 expression levels (18). We confirmed this in current analysis, as the ICER was $69182.50 in advanced NSCLC patients with a high TMB, irrespective of their tumor PD-L1 expression levels, which was lower than the values of ICER in three PD-L1 expression populations (≥50, ≥1, and <1%). Moreover, the results of probabilistic sensitivity analyses revealed an 81.1% chance of nivolumab plus ipilimumab vs. chemotherapy being cost-effective in patients with a high TMB, which is higher than those of the three PD-L1 expression populations (≥50, ≥1, and <1%). Despite that nivolumab plus ipilimumab provided the greatest absolute survival for patients with a high TMB in CheckMate 227, yet the clinical benefits of nivolumab plus ipilimumab and those of chemotherapy were similar in patients regardless of their TMB. The unexpected impacts of TMB on the overall survival of patients receiving chemotherapy may be the cause of these results (41–45). Thus, the benefits of nivolumab plus ipilimumab may be overestimated or underestimated in our analysis. Therefore, it is necessary to understand the functions of TMB as a biomarker before including it into clinical practice.

Pembrolizumab monotherapy, pembrolizumab plus chemotherapy and atezolizumab combined with bevacizumab and chemotherapy are the first-line immunotherapy in advanced NSCLC. The following are some related cost-effectiveness analyses based on cases in the US. Atezolizumab combined with bevacizumab and chemotherapy was not a cost-effective choice for patients with advanced NSCLC (46). In contrast, pembrolizumab plus chemotherapy was estimated to be cost-effective in advanced NSCLC from the US payers' view (47, 48). Pembrolizumab monotherapy for patients with PD-L1 ≥ 1% is the only chemotherapy-spared therapy approved by US FDA for the first-line treatment of advanced NSCLC patients based on the trial of KEYNOTE-042 (10). The cost-effectiveness analysis of KEYNOTE-042 displayed that ICERs of $136228.82, $160625.98, and $179530.17 per QALY for advanced NSCLC patients with PD-L1 ≥50, ≥20, and ≥1%, respectively, and that pembrolizumab monotherapy was cost-effective in patients with PD-L1 ≥ 50% but not in the ≥20 and 1% populations at a WTP threshold of $150,000 per QALY in the US (49). In our analysis, it estimated that compared with chemotherapy, nivolumab plus ipilimumab was cost-effective in advanced NSCLC patients with PD-L1 ≥ 50% and PD-L1 ≥ 1% but not in the PD-L1 <1% populations at a WTP threshold of $150,000 per QALY. It means that the indication of nivolumab plus ipilimumab has expanded compared with that of PD-1 inhibitor monotherapy alone. Moreover, treatment with nivolumab plus ipilimumab is a cost-effective choice for patients with PD-L1 ≥ 1% who desire chemotherapy-free treatment. In addition, regardless of the PD-L1 expression levels, nivolumab plus ipilimumab was cost-effective in patients with a high TMB. However, due to the absence of head-to-head trials of pembrolizumab vs. nivolumab plus ipilimumab, caution should be in place before drawing any conclusion of which treatment would be more cost-effective.

Our model estimated the cost and effect over the entire runtime of the model, and then obtained results. However, we noted that the survival curves of the nivolumab plus ipilimumab group and the chemotherapy group started to cross ~6 months after treatment, which indicated that the efficacy of the chemotherapy group was better than that of the nivolumab plus ipilimumab group within the first 6 months. It was consistent with the survival data provided by KEYNOTE-042 (10, 50). This may indicate that patients receiving nivolumab plus ipilimumab treatment, if not gaining benefits from combination immunotherapy, would progress rapidly or die within 6 months of treatment. These statistics suggest that in unsegmented populations, these patients receiving immunotherapy are likely to risk rapid progress or death. This may lead our model to overestimate the benefits of treatment with nivolumab plus ipilimumab in this treatment period.

We also discovered that the mortality risk in patients with PD-L1 expression of 1–49% had no statistical significance (hazard ratio 0.94, 95% CI 0.75–1.18) when compared to that in the PD-L1 ≥ 50% (hazard ratio 0.70, 95% CI 0.55–0.90) and ≥1% (hazard ratio 0.79, 95% CI 0.65–0.96) populations. And further analysis has found that the populations with PD-L1 ≥ 1% did not exclude the PD-L1 ≥ 50% populations, and that the majority of benefits in the former were manifested in the latter. Thus, it may overestimate the cost-effectiveness of benefits of nivolumab plus ipilimumab in patients with PD-L1 expression of 1–49%. Careful deliberation is called for should these patients be to use combination immunotherapy. Furthermore, patients with PD-L1 expression of 1–49% were not estimated in our analysis, because the trial of CheckMate 227 did not report enough survival data on these patients.

Like any other models, there are some limitations in our analysis. First, the survival data shows a dramatic tail on the OS curve in CheckMate 227, and the benefits in the dual checkpoint inhibition group will become more significant when compared to those of the chemotherapy with a long-term follow-up. It may underestimate the benefits of combined immunotherapy because our study is based on data of a 29.3-months follow-up. Moreover, our model adds too much weight to PFS and PD. However, most cost-effectiveness analyses of immune checkpoint inhibitors were based on Markov model (31, 34, 46, 51). Thus, the results of the present study should be interpreted with discretion, especially those of the PD-L1 <1% populations. Second, immunotherapy-related AEs are rare, and the cost of treatment in such cases is rather high. Therefore, more cases of immunotherapy-related AEs would be conducive to more accurate evaluation of AE cost for patients using nivolumab plus ipilimumab. Besides, the benefits of nivolumab plus ipilimumab would be overestimated in this model. Third, considering the model hypothesis, the exploratory nature of the subgroup analyses and the small sample subgroup size, the results of the subgroup analyses in the current study should be analyzed with caution. Fourth, standard data was commonly used in our model to estimate drug dose, and it should be adjusted according to the patients' physical conditions which may generate bias.

## Conclusion

When compared to chemotherapy, nivolumab plus ipilimumab as first-line treatment is cost-effective in advanced NSCLC patients with PD-L1 ≥ 50%, PD-L1 ≥ 1% or a high TMB but is not cost-effective in PD-L1 <1% population in view of US payers.

## Data Availability Statement

The original contributions presented in the study are included in the article/Supplementary Material, further inquiries can be directed to the corresponding author/s.

## Ethics Statement

The current research was underlain by model techniques and literature reviews; no written consent was required as per the ethics committee of the Xiangya Hospital of Central South University (Changsha, People's Republic of China).

## Author Contributions

HH, LS, and JH conceived and designed the experiments. HH, LS, LY, and DD performed the experiments. HH, LS, LY, DD, JH, YS, and ML analyzed the data. SZ, JH, and DC contributed reagents, materials, and analysis tools. HH, LS, and JH wrote the manuscript. All authors read and approved the final manuscript. All authors contributed to the article and approved the submitted version.

## Conflict of Interest

The authors declare that the research was conducted in the absence of any commercial or financial relationships that could be construed as a potential conflict of interest.

## Supplementary Material

Supplementary Figure 1 Markov states. To illustrate the disease development process of advanced non-small cell lung cancer (NSCLC).

Supplementary Figure 2 The results of Monte Carlo probabilistic sensitivity analysis. **(A)** Nivolumab plus ipilimumab vs. chemotherapy in programmed death ligand 1 (PD-L1) ≥ 50% population. **(B)** Nivolumab plus ipilimumab vs. chemotherapy in PD-L1 ≥ 1% population. **(C)** Nivolumab plus ipilimumab vs. chemotherapy in PD-L1 <1% population.

Supplementary Figure 3 The results of Monte Carlo probabilistic sensitivity analysis of nivolumab plus ipilimumab vs. chemotherapy in patients with high tumor mutational burden (TMB).
