# Supplementary figures and images for: Cost-Effectiveness Analysis of Nivolumab Plus Ipilimumab vs. Chemotherapy as First-Line Therapy in Advanced Non-Small Cell Lung Cancer
Source: Front Oncol. 2020 Sep 8;10:1649. doi: 10.3389/fonc.2020.01649 (PMC7507990; doi:10.3389/fonc.2020.01649)

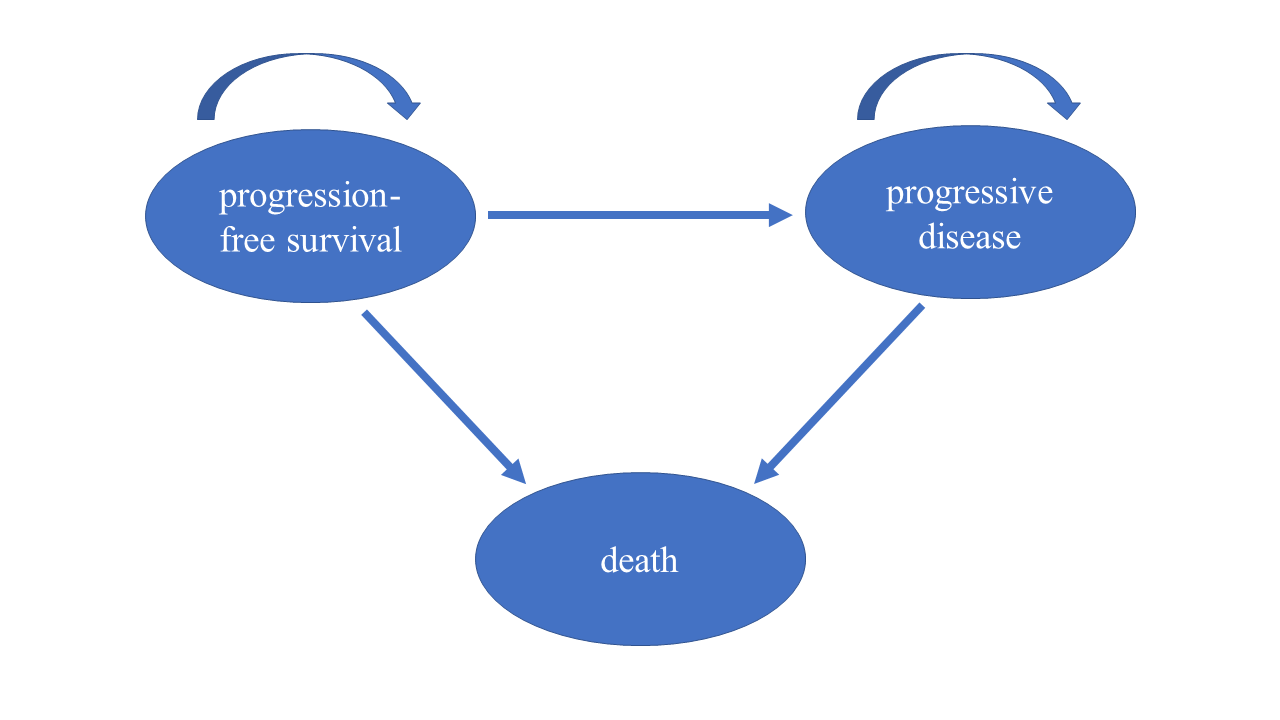

Supplement: Supplementary Figure 1 — Markov states. To illustrate the disease development process of advanced non-small cell lung cancer (NSCLC). [file Image_1.png]

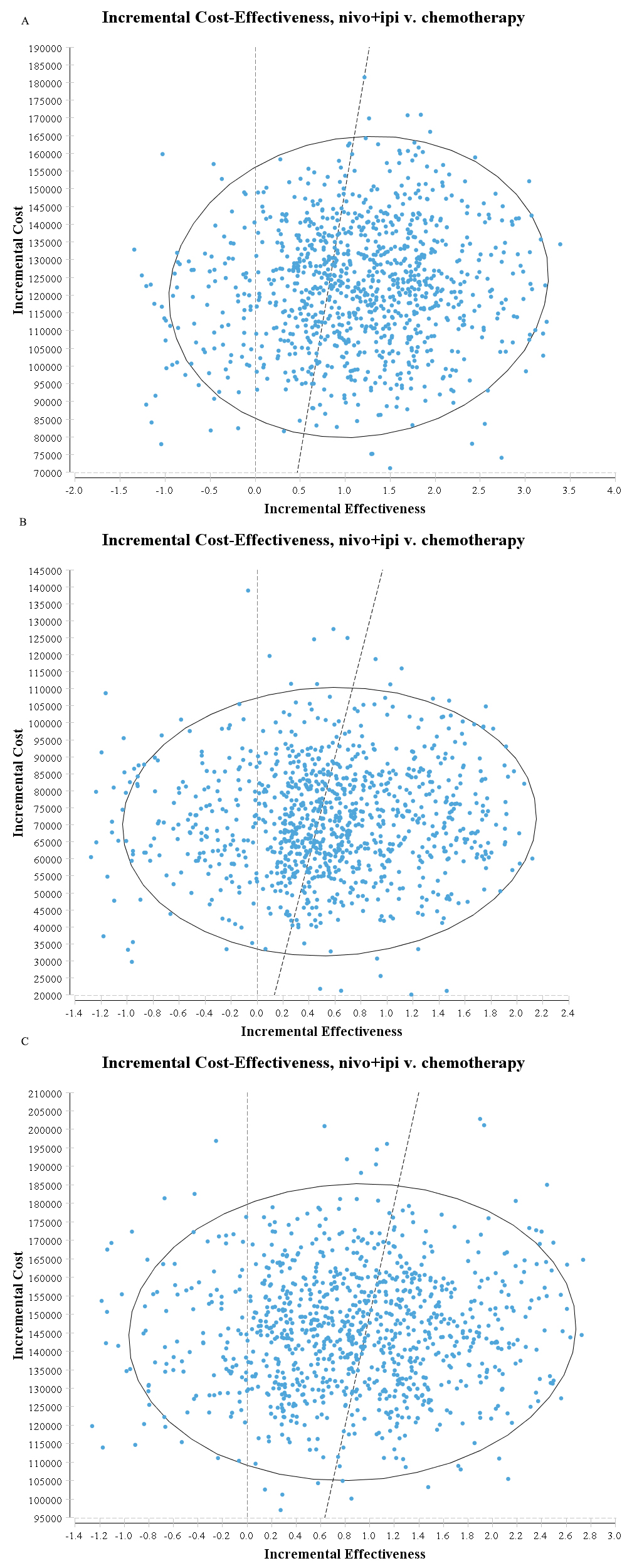

Supplement: Supplementary Figure 2 — The results of Monte Carlo probabilistic sensitivity analysis. (A) Nivolumab plus ipilimumab vs. chemotherapy in programmed death ligand 1 (PD-L1) ≥ 50% population. (B) Nivolumab plus ipilimumab vs. chemotherapy in PD-L1 ≥ 1% population. (C) Nivolumab plus ipilimumab vs. chemotherapy in PD-L1 <1% population. [file Image_2.JPEG]

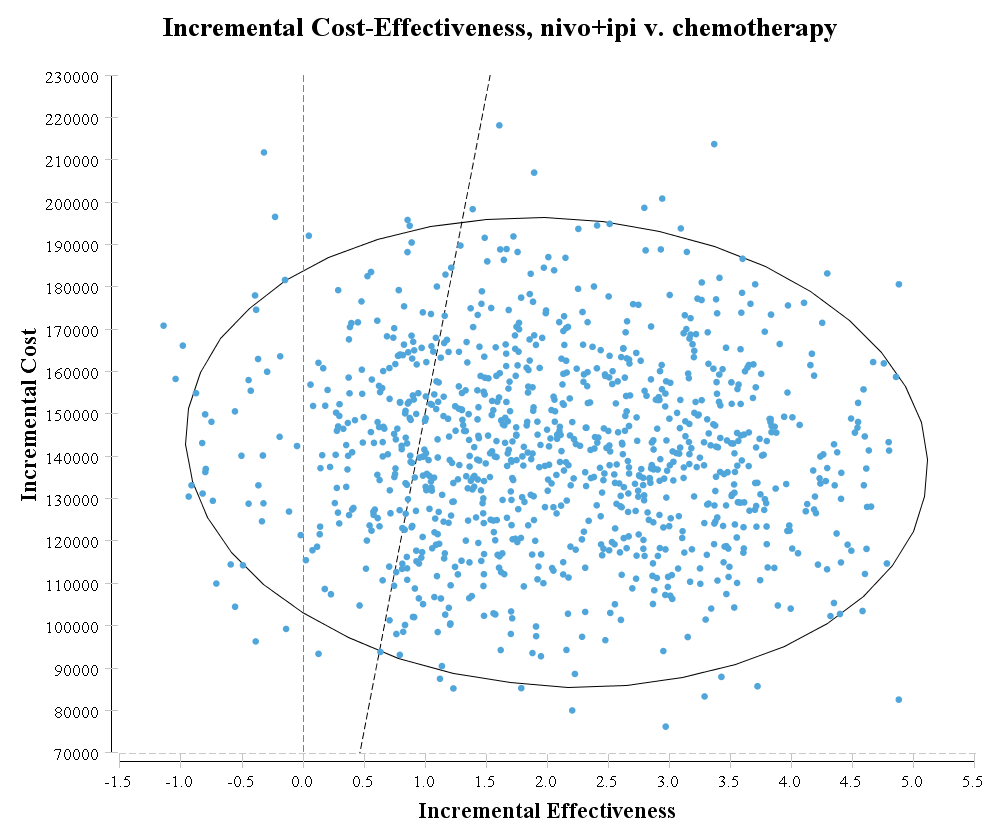

Supplement: Supplementary Figure 3 — The results of Monte Carlo probabilistic sensitivity analysis of nivolumab plus ipilimumab vs. chemotherapy in patients with high tumor mutational burden (TMB). [file Image_3.PNG]
