# Supplementary material for: Cost-Effectiveness Analysis of Nivolumab Plus Ipilimumab vs. Chemotherapy as First-Line Therapy in Advanced Non-Small Cell Lung Cancer
Source: Front Oncol. 2020 Sep 8;10:1649. doi: 10.3389/fonc.2020.01649 (PMC7507990; doi:10.3389/fonc.2020.01649)
Supplement: Supplementary file 4 [file Table_1.docx]

**Supporting Table 1. Results of subgroup analysis in PD-L1 expression ≥1% populations.**

| **Subgroup** | **HR for OS (95% CI)** | **ICER per QALY (95%CI), $** | **Cost-effectiveness probability at WTP $150 000/QALY** |
| --- | --- | --- | --- |
| **Age** |  |  |  |
| <65 years | 0.70 (0.55–0.89) | 124555 (120561–129057) | 57.4% |
| 65 to 75 years | 0.91 (0.70–1.19) | 129477 (124555–133554) | 54.7% |
| ≥75 years | 0.92 (0.57–1.48) | 129681 (121107–130957) | 54.3% |
| **Sex** |  |  |  |
| Male | 0.75 (0.61–0.93) | 125817 (122188–129883) | 56.4% |
| Female | 0.91 (0.69–1.21) | 129477 (124298–133671) | 54.7% |
| **ECOG score** |  |  |  |
| 0 | 0.66 (0.48–0.89) | 123517 (118631–129057) | 58.9% |
| 1 | 0.89 (0.73–1.09) | 129057 (125318–132559) | 55.2% |
| **Smoking status** |  |  |  |
| Never smoked | 1.23 (0.76–1.98) | 133756 (126063– -73429) | 53.8% |
| Current or former smoker | 0.77 (0.64–0.92) | 126308 (122989–129681) | 56.1% |
| **Tumor histologic type** |  |  |  |
| Squamous | 0.69 (0.52–0.92) | 124298 (119737–129681) | 58.2% |
| Nonsquamous | 0.85 (0.69–1.04) | 128183 (124298–131846) | 55.5% |
| **Liver metastatic** |  |  |  |
| Yes | 1.05 (0.74–1.49) | 131999 (125568–130629) | 54.2% |
| No | 0.76 (0.63–0.92) | 126063 (122723–129681) | 56.3% |
| **Bone metastatic** |  |  |  |
| Yes | 0.75 (0.55–1.03) | 125817 (120561–131689) | 56.4% |
| No | 0.81 (0.67–0.99) | 127265 (123779–131015) | 55.9% |
| **CNS metastatic** |  |  |  |
| Yes | 0.68 (0.41–1.11) | 124039 (116688–132807) | 58.5% |
| No | 0.82 (0.68–0.98) | 127498 (124039–130835) | 55.4% |

Abbreviation: CI, confidence interval; CNS, central nervous system; ECOG, Eastern Cooperative Oncology Group; HR, hazard ratio; ICER, incremental cost-effectiveness ratio; OS, overall survival; PD-L1, programmed death ligand 1; QALY, quality-adjusted life-year; WTP, willingness-to-pay.
