# Supplementary material for: Cost-Effectiveness Analysis of Nivolumab Plus Ipilimumab vs. Chemotherapy as First-Line Therapy in Advanced Non-Small Cell Lung Cancer
Source: Front Oncol. 2020 Sep 8;10:1649. doi: 10.3389/fonc.2020.01649 (PMC7507990; doi:10.3389/fonc.2020.01649)
Supplement: Supplementary file 5 [file Table_2.docx]

**Supporting Table 2. Results of subgroup analysis in PD-L1 expression <1% populations.**

| **Subgroup** | **HR for OS (95% CI)** | **ICER per QALY (95%CI), $** | **Cost-effectiveness probability at WTP $150 000/QALY** |
| --- | --- | --- | --- |
| **Age** |  |  |  |
| <65 years | 0.69 (0.50–0.94) | 143880 (133506–158711) | 49.7% |
| 65 to 75 years | 0.49 (0.32–0.75) | 132983 (124509–147320) | 54.8% |
| ≥75 years | 0.75 (0.31–1.82) | 147320 (124037–233602) | 46.8% |
| **Sex** |  |  |  |
| Male | 0.55 (0.41–0.73) | 136155 (128896–146165) | 53.8% |
| Female | 0.83 (0.54–1.28) | 152024 (135620–181391) | 50.3% |
| **ECOG score** |  |  |  |
| 0 | 0.78 (0.50–1.23) | 149068 (133506–177811) | 46.4% |
| 1 | 0.55 (0.42–0.74) | 136155 (129398–146741) | 53.8% |
| **Smoking status** |  |  |  |
| Never smoked | 0.60 (0.32–1.15) | 138863 (124509–172287) | 52.1% |
| Smoked | 0.63 (0.49–0.82) | 140516 (132983–151428) | 53.5% |
| **Tumor histologic type** |  |  |  |
| Squamous | 0.49 (0.30–0.79) | 132983 (123567–149655) | 54.8% |
| Nonsquamous | 0.67 (0.51–0.88) | 142750 (134031–155031) | 50.0% |
| **Liver metastatic** |  |  |  |
| Yes | 0.52 (0.32–0.83) | 134558 (124509–152024) | 53.4% |
| No | 0.65 (0.49–0.86) | 141628 (132983–153822) | 49.2% |
| **Bone metastatic** |  |  |  |
| Yes | 0.58 (0.37–0.89) | 137773 (126918–155639) | 53.1% |
| No | 0.64 (0.48–0.85) | 141071 (132463–153220) | 52.0% |
| **CNS metastatic** |  |  |  |
| Yes | 0.54 (0.24–1.22) | 135620 (120815–177108) | 52.5% |
| No | 0.62 (0.48–0.80) | 139963 (132463–150244) | 48.8% |

Abbreviation: CI, confidence interval; CNS, central nervous system; ECOG, Eastern Cooperative Oncology Group; HR, hazard ratio; ICER, incremental cost-effectiveness ratio; OS, overall survival; PD-L1, programmed death ligand 1; QALY, quality-adjusted life-year; WTP, willingness-to-pay.
