# Supplementary material for: Cost-Effectiveness Analysis of Nivolumab Plus Ipilimumab vs. Chemotherapy as First-Line Therapy in Advanced Non-Small Cell Lung Cancer
Source: Front Oncol. 2020 Sep 8;10:1649. doi: 10.3389/fonc.2020.01649 (PMC7507990; doi:10.3389/fonc.2020.01649)
Supplement: Supplementary file 6 [file Table_3.docx]

Supporting Table 3. Model parameters: baseline values, ranges, and distributions for sensitivity analysis in patients with high tumor mutational burden.

| **Variable** | **Baseline value** | **Range** | | **Reference** | **Distribution** |
| --- | --- | --- | --- | --- | --- |
|  |  | **Minimum** | **Maximum** |  |  |
| **Weibull survival model in nivolumab plus ipilimumab group with high TMB** |  |  |  |  |  |
| PFS | Shape=0.539682, Scale=0.269634 | - | - | (17, 18) | - |
| OS | Shape=0.545941, Scale=0.106556 | - | - | (17, 18) | - |
| **Weibull survival model in chemotherapy group with high TMB** |  |  |  |  |  |
| PFS | Shape=1.30290, Scale=0.13932 | - | - | (17, 18) | - |
| OS | Shape=0.949337, Scale=0.058441 | - | - | (17, 18) | - |
| **Proportion of tumor histologic type in chemotherapy** |  |  |  |  |  |
| **High TMB** |  |  |  |  |  |
| Nonsquamous | 65.6 | - | - | (17, 18) | - |
| Squamous | 34.4 | - | - | (17, 18) | - |
| **Proportion of treatment discontinuation** |  |  |  |  |  |
| **High TMB** |  |  |  |  |  |
| Nivolumab plus ipilimumab | 0.165 | - | - | (17, 18) | - |
| Chemotherapy | 0.431 | - | - | (17, 18) | - |
| **Risk for main adverse events in nivolumab plus ipilimumab group with high TMB** |  |  |  |  |  |
| Risk of anemia | 0.02 | 0.016 | 0.024 | (17, 18) | Beta |
| Risk of rash | 0.02 | 0.016 | 0.024 | (17, 18) | Beta |
| Risk of nausea | 0.01 | 0.008 | 0.012 | (17, 18) | Beta |
| **Risk for main adverse events in chemotherapy group with high TMB** |  |  |  |  |  |
| Risk of anemia | 0.08 | 0.064 | 0.096 | (17, 18) | Beta |
| Risk of neutropenia | 0.12 | 0.096 | 0.144 | (17, 18) | Beta |
| Risk of nausea | 0.01 | 0.008 | 0.012 | (17, 18) | Beta |
| Risk of neutrophil count decreased | 0.06 | 0.048 | 0.072 | (17, 18) | Beta |
| Risk of decreased appetite | 0.03 | 0.024 | 0.036 | (17, 18) | Beta |
| Risk of vomiting | 0.04 | 0.032 | 0.048 | (17, 18) | Beta |
| Risk of diarrhea | 0.01 | 0.008 | 0.012 | (17, 18) | Beta |
| **Nivolumab plus ipilimumab group subsequent therapy proportion in high TMB population** |  |  |  |  |  |
| Nivolumab | 0.022 | - | - | (17, 18) | - |
| Chemotherapy | 0.014 | - | - | (17, 18) | - |
| Targeted therapy | 0.158 | - | - | (17, 18) | - |
| **Chemotherapy group subsequent therapy proportion in high TMB population** |  |  |  |  |  |
| Nivolumab | 0.225 | - | - | (17, 18) | - |
| Pembrolizumab | 0.038 | - | - | (17, 18) | - |
| Ipilimumab | 0.031 | - | - | (17, 18) | - |
| Targeted therapy | 0.019 | - | - | (17, 18) | - |
| Chemotherapy | 0.206 |  |  | (17, 18) |  |
| **Utility** |  |  |  |  |  |
| Utility PFS in nivolumab plus ipilimumab | 0.784 | 0.74 | 0.828 | (19) | Beta |
| Utility PFS in chemotherapy | 0.693 | 0.642 | 0.743 | (19) | Beta |
| Utility progressive disease | 0.473 | 0.166 | 0.568 | (22) | Beta |
| **Patients’ weight, kg** | 70 | - | - | (23) | Beta |
| **Body surface area, m^2^** | 1.84 | - | - | (23) | Beta |
| **Drug cost, $/per cycle** |  |  |  |  |  |
| Nivolumab | 17517.15 | 14013.72 | 21020.58 | (17, 18, 24) | Gamma |
| Ipilimumab | 10718.96 | 8575.17 | 12862.75 | (17, 18, 24) | Gamma |
| Pemetrexed | 12782.85 | 10226.28 | 15339.42 | (17, 18, 24) | Gamma |
| Gemcitabine | 92 | 73.6 | 110.4 | (17, 18, 24) | Gamma |
| Carboplatin | 56.55 | 45.24 | 67.86 | (17, 18, 24) | Gamma |
| Cisplatin | 51.78 | 41.42 | 62.14 | (17, 18, 24) | Gamma |
| Pembrolizumab | 19755.6 | 15804.48‬ | 23706.72‬ | (17, 18, 24) | Gamma |
| Targeted therapy | 12615.68 | 10092.54 | 15138.82 | (17, 18, 24) | Gamma |
| Subsequent chemotherapy | 238.74 | 190.99 | 286.49 | (17, 18, 24) | Gamma |
| **Expenditures on main adverse events, $** |  |  |  |  |  |
| Anemia | 7969.56 | 6375.65 | 9536.47 | (30) | Gamma |
| Neutropenia | 32995 | 24746 | 41244 | (31) | Gamma |
| Neutrophil count decreased | 32995 | 24746 | 41244 | (31) | Gamma |
| Rash | 13376 | 10700.8 | 16051.2 | (33) | Gamma |
| Diarrhea | 10301 | 8240.8 | 12361.2 | (33) | Gamma |
| Decreased appetite | 9711 | 7768.8 | 11653.2 | (33) | Gamma |
| Vomiting | 10301 | 8240.8 | 12361.2 | (33) | Gamma |
| Nausea | 10301 | 8240.8 | 12361.2 | (33) | Gamma |
| **Administration $/per cycle** | 139.61 | 111.69 | 167.53 | (25) | Gamma |
| **CT $/per cycle** | 231 | 208 | 254 | (28) | Gamma |
| **Laboratory $/per cycle** | 315 | 252 | 378 | (29) | Gamma |
| **Discount rate** | 0.03 | - | - | (20) | - |

Abbreviation: CT, compute tomography; NSCLC, non-small-cell lung cancer; OS, overall survival; PFS, progression-free survival; TMB, tumor mutational burden.
