# Supplementary material for: Cost-Effectiveness Analysis of Nivolumab Plus Ipilimumab vs. Chemotherapy as First-Line Therapy in Advanced Non-Small Cell Lung Cancer
Source: Front Oncol. 2020 Sep 8;10:1649. doi: 10.3389/fonc.2020.01649 (PMC7507990; doi:10.3389/fonc.2020.01649)
Supplement: Supplementary file 7 [file Table_4.docx]

**Supporting Table 4. Baseline results in nivolumab plus ipilimumab and chemotherapy groups in patients with high tumor mutational burden.**

| **Strategies and Scenarios** | **Total cost, $** | **LYs** | **QALYs** | **ICER per LY ^a^** | **ICER per QALY ^b^** |
| --- | --- | --- | --- | --- | --- |
| High TMB |  |  |  |  |  |
| Nivolumab plus ipilimumab | 404241.67 | 6.84 | 3.90 | 39864.15 | 69182.50 |
| Chemotherapy | 262985.94 | 3.30 | 1.86 | - | - |

a, Compared to chemotherapy ($/LY); b, Compared to chemotherapy ($/QALY).

Abbreviation: ICER, incremental cost-effectiveness ratio; LY, life-year; QALY, quality-adjusted life-year; TMB, tumor mutational burden.
